# Beclin-1 is a novel predictive biomarker for canine cutaneous and subcutaneous mast cell tumors

**DOI:** 10.1177/03009858211042578

**Published:** 2021-09-14

**Authors:** Britta J. Knight, Geoffrey A. Wood, Robert A. Foster, Brenda L. Coomber

**Affiliations:** 1 Department of Pathobiology, Ontario Veterinary College, University of Guelph, Guelph, Ontario, Canada; 2 Department of Biomedical Sciences, Ontario Veterinary College, University of Guelph, Guelph, Ontario, Canada

**Keywords:** beclin-1, dog, mast cell tumor, predictive biomarker, survival, tissue microarray

## Abstract

Mast cell tumors (MCTs) are the most common skin tumor of the dog, and accurately predicting their clinical behavior is critical in directing patient therapy, as they range from benign lesions to a fatal systemic disease. Grading is useful for prognosis, but it cannot predict the behavior of all MCTs. We hypothesized that biomarker immunolabeling in tumor tissues would correlate with patient morbidity and mortality. A clinically annotated tissue microarray (TMA) of primary, recurrent, and metastatic (to lymph node) canine dermal and subcutaneous MCTs was created. Some dogs whose MCTs were included in the TMA did not receive adjunctive treatment after surgical excision of the MCT, whereas others were treated with one or a combination of chemotherapy, radiation, or oral toceranib. Immunohistochemistry for beclin-1, an autophagy protein, was performed followed by digital image analysis. Beclin-1 immunolabeling was higher in recurrent tumors (mean *H*-score 110.8) than primary MCTs (mean *H*-score 73.5), and highest in lymph node metastases (mean *H*-score 138.5) with a significant difference in means (*P* < .001). While beclin-1 level was not prognostic, it was strongly predictive for survival after adjunctive treatment; dogs with high beclin-1-expressing tumors showed poorer survival compared to those with low beclin-1-expressing tumors (HR = 5.7, *P* = .02), especially in Kiupel high-grade tumors (HR = 16.3, *P* = .01). Beclin-1 immunolabeling was the only significant predictive factor by multivariable analysis (*P* = .04). These findings may improve our ability to predict the response to adjunctive therapy. Importantly, these data suggest that autophagy inhibitors may be useful in improving response to treatment for dogs with high-grade MCTs.

Canine dermal and subcutaneous mast cell tumors (MCTs) are the most common skin tumor of the dog, representing up to 21% of all skin tumors.^4,7,25,51^ Skin MCTs arise in either the dermis or the subcutaneous tissue, at a ratio of approximately 6:1.^37^ The behavior of MCTs can vary widely, with many of the tumors having a favorable prognosis, and fewer MCTs developing local recurrence and/or metastases to the draining lymph node or disseminated throughout the body, which typically includes, but is not limited to, spleen and liver.^28^

The ability to accurately predict the behavior of a dermal and subcutaneous MCT is critical in directing patient therapy. Biomarkers are measured variables that are associated with disease outcome. Prognostic biomarkers are associated with disease outcome independent of treatment, whereas predictive biomarkers inform about treatment effect.^1^ Prognostic biomarkers may also inform about disease outcome in patients who do not receive adjunctive treatment. In the case of MCTs, a prognostic biomarker that can predict the natural progression of disease after surgical removal of a tumor would be particularly useful to assist clinicians and owners in deciding whether to pursue additional adjunctive therapy.

Efforts to improve prognostication have been made through characterization of the receptor tyrosine kinase (RTK), KIT. Canine normal and neoplastic mast cells typically display 1 of 3 distinct KIT immunolabeling patterns, namely, membranous (pattern I), focal/stippled cytoplasmic (pattern II), and diffuse cytoplasmic (pattern III). Normal mast cells show membranous KIT immunolabeling, whereas neoplastic cells can show any one of the 3 patterns.^48^ Cytoplasmic KIT protein localization (patterns II and III) is significantly associated with increased tumor recurrence and reduced survival,^20,43,49^ higher histological grade and increased cell proliferation^13^ in dermal MCTs, and increased recurrence and metastases in subcutaneous MCTs.^44^ Elevated expression of the cellular proliferation marker Ki67 and the proapoptotic protein BAX are also negative prognostic factors.^10^

Although KIT immunolabeling pattern, Ki67, and BAX are prognostic, the most reliable prognostic indicators currently used for dermal MCTs are 2 histologic grading schemes. The first widely used grading scheme was the Patnaik grading scheme,^32^ which was developed for dermal MCTs. This scheme compared histomorphologic features to define 3 grades of tumors. Grade I tumors include well-differentiated tumors that are confined to the dermis with no observed mitotic figures in the section examined; grade II tumors include intermediately differentiated tumors infiltrating or replacing the lower dermal and subcutaneous tissue, with 0 to 2 mitotic figures per 400× high power field (HPF); and grade III tumors include highly cellular, poorly differentiated tumors with replacement of subcutaneous and deep tissues, and 3 to 6 mitotic figures per HPF. This scheme was able to separate clinically benign from highly aggressive tumors, as evidenced by the fact that grade I tumors showed much higher survival rates (93% alive at 1500 days) compared with grade III tumors (6% alive at 1500 days). The second widely used grading scheme is the Kiupel grading scheme.^19^ This grading scheme is a 2-tier system that separates low-grade versus high-grade MCTs based on mitotic count, presence of multinucleated cells, bizarre nuclei, and/or karyomegaly. The most useful prognostic indicator for subcutaneous MCTs is the mitotic count.^44^ Although much progress was made in improving prognostication of MCTs, both current schemes have limitations. For example, in the Patnaik grading scheme, the majority of MCTs fall into the grade II category, whose behavior is difficult to predict.^37,41^ In addition, approximately 5% to 15% of dogs with Kiupel low-grade MCTs (which represented 76% to 90% of the studied tumors) eventually died or were euthanized due to MCT-related disease,^19,41^ suggesting some inconsistency between pathological grade and clinical behavior.

In addition to the development of accurate prognostic markers for both human and animal cancer, there has also been tremendous effort to develop accurate markers to predict therapeutic response. The only predictive marker that has been published to date in canine MCT is c-KIT mutational status. However, since MCTs with both mutated and wildtype c-KIT respond to treatment with RTK inhibitors,^15,24,50^ mutation analysis has low clinical utility. Given the limitations of the current grading systems, we sought to identify a novel prognostic and/or predictive biomarker for canine dermal and subcutaneous MCTs.

Autophagy is a homeostatic mechanism operating at low basal levels that enables targeted destruction of damaged proteins and organelles. To survive times of adverse microenvironmental conditions, such as nutrient starvation or growth factor depletion, cells can undergo autophagy to degrade and recycle cellular components to maintain a source of molecular substrates.^3^ The process of autophagy begins with nucleation, whereby multiple proteins assemble to form a phagophore.^3^ The walls of the phagophore join to form the autophagosome, which then fuses with the lysosome. Cancer cells are also able to exploit the process of autophagy in order to survive the poor conditions in the tumor microenvironment.^30^ The protein beclin-1 is encoded by the autophagy related gene, BECN1, and plays a key role in the nucleation step of autophagy.^27^

The aim of this study was to investigate beclin-1 immunolabeling levels as a prognostic (informing on natural disease progression after surgical removal) and/or predictive marker in canine dermal and subcutaneous MCTs, and compare its utility to the current prognostic features.

## Materials and Methods

Paraffin-embedded tissue blocks and hematoxylin and eosin–stained slides were collected from the Animal Health Laboratory (exclusively from the Ontario Veterinary College Health Sciences Centre, University of Guelph, Guelph, Ontario, Canada), Yager-Best Histovet Histological and Cytological Services (Guelph, Ontario, Canada), and Antech Diagnostics Canada (Mississauga, Ontario, Canada). The 139 MCTs (83 dermal, 43 subcutaneous, 13 lymph node metastases) from 97 dogs from the Ontario Veterinary College were from 2008 to 2017, the 56 MCTs (all subcutaneous) from 55 dogs from Yager-Best were from 2002 to 2006, and the 34 MCTs (27 dermal and 7 subcutaneous) from 30 dogs from Antech were from 2014. The tissue blocks and medical records for the Yager-Best cases had been collected for a previous study.^44^ For the Ontario Veterinary College cases, the medical records were reviewed to obtain the breed, sex, date of diagnosis, tumor site, details of previous cancer including MCTs, adjunctive treatment protocols, metastatic disease status, date of euthanasia/death, and cause of death. If full outcome data were not available from the medical record, the referring clinic was contacted directly to obtain these data. When histology or fine needle aspirates were not done, the presence of local recurrence and metastasis was suspected based on physical examination. The clinical data and raw data are in Supplemental Tables S1 and S7.

The date of diagnosis was defined as the date of MCT surgical excision. Disease-free interval (DFI) was defined as the number of days from the date of diagnosis to confirmation (histology or fine needle aspiration) or suspicion (clinical signs) of local recurrence of the MCT or metastasis (lymph node metastasis or disseminated MCT disease in internal organs and/or skin). Local recurrence was defined as regrowth at the site of surgical excision. Survival time (ST) was defined as the number of days from the date of diagnosis to euthanasia/death. The cause of death was recorded as being MCT-related (euthanasia due to local recurrence affecting quality of life or widespread metastases) or unrelated to MCT. Follow-up survival time ranged from 15 days to 7 years. Outcome data are summarized in Supplemental Table S2. For the survival analyses, cases were included if there was an attempt to surgically remove the dermal or subcutaneous MCT (regardless of the size of the surgical margins as measured by radial sectioning and histopathology) and included both primary occurrences and recurrences. For the analysis of beclin-1 levels in tumors that recurred after adjunctive therapy, there was one dog with 2 new growths that developed near the site of the primary MCT that were considered recurrences, and these along with the primary MCT were included in the tissue microarray (TMA); all other recurrences did not have the paired primary MCT available for inclusion in the TMA. The TMA was constructed using a Pathology Devices TMArrayer (Pathology Devices).

For all tumors, hematoxylin and eosin–stained sections were examined and type of MCT, Patnaik grade, Kiupel grade, and mitotic count were determined through a consensus reached by 2 pathologists (BK and RF). The Patnaik grade was determined for dermal MCTs only, as subcutaneous invasion is a feature in the grading scheme and Kiupel grade was determined for both dermal and subcutaneous MCTs, as at the time of publication of this grading scheme, many pathologists did not distinguish between dermal and subcutaneous MCTs. The mitotic count (number per 10 high-power fields with FN of 22 mm [2.37 mm^2^]) was determined on the original section and performed in the most mitotically active area. The pathologists were not aware of previous grading or patient outcome at the time of slide evaluation. In a study examining only subcutaneous MCTs, mitotic count was the only parameter found to be a predictor of survival.^44^ Therefore, in order to directly compare dermal and subcutaneous MCTs by mitotic count, both categories of tumors were divided into 2 groups based on the mitotic count in the area of the highest mitotic activity, and the cutoffs established in for subcutaneous tumors. A “low mitotic count” defined as a mitotic count of 4 or less in ten 400× fields, and a “high mitotic count” as a mitotic count of 5 or greater in ten 400× fields. Three 6-mm-diameter cores were taken from each tumor, unless the tumor area was less than approximately 25 mm^2^, in which case 1 or 2 cores were taken.

Immunohistochemistry was run within 7 days of the slides being sectioned. Unstained tissue sections were baked in the oven overnight at 37 °C, deparaffinized in xylene, and rehydrated. Heat-based antigen retrieval was performed using a Biocare Medical Decloaking Chamber NxGen Model: DC2012 (Biocare Medical) by incubating the sections in sodium citrate pH 6 at 110 °C for 5 minutes and then allowed to cool to 80 °C. The sections were incubated with DAKO Peroxidase Blocking Reagent (DAKO Corporation) for 5 minutes at room temperature, and then incubated with at 1:300 dilution with anti-beclin-1 antibody (mouse monoclonal antibody, LS-C172820, LSBio) or at 1:200 dilution with anti-KIT antibody (rabbit polyclonal, A4502, DAKO) overnight at 4 °C. The next day, the sections were incubated with DAKO Envision secondary anti-mouse antibody (DAKO Corporation) for 30 minutes at room temperature. After washing with TBST, the sections were incubated with 3,3′-diaminobenzidine (DAB) chromogen for 10 minutes and counterstained with Harris modified hematoxylin. The antibody for beclin-1 has been verified in canine tissues by western blot.^39^ Each TMA block included tissue spots of canine renal tubular epithelium as a positive control tissue for beclin-1 (Suppl. Fig. S1)^21,47^ and canine cerebellum as a positive control tissue for KIT (Suppl. Fig. S2).^35^ A mouse monoclonal IgG2a isotype antibody (61656S, Cell signaling; Suppl. Fig. S3) and a rabbit IgG, whole molecule antibody (011-000-003, Jackson; Suppl. Fig. S4) were used in place of the primary antibodies as isotype controls for beclin-1 and KIT, respectively. The slides were scanned using the Leica SCN400 Slide Scanner automated digital image system (Leica Microsystems) by the Digital Histology Shared Resource at Vanderbilt University Medical Center, Nashville, Tennessee. The whole slide images were scanned at 20× magnification at a resolution of 0.5 µm/pixel. The tissue cores were mapped using Ariol Review software within the Digital Histology Shared Resource (https://www.vumc.org/dhsr/welcome).

All tissue spots of immunolabeled slides were evaluated manually to ensure that they accurately represented the targeted sample region, and for quality of the spot and quality of the digital image. Slides immunolabeled with KIT were evaluated by a single pathologist (BK) to determine the KIT immunolabeling pattern for each tumor (1 = perimembranous, 2 = focal or stippled, 3 = diffuse). If the tissue spot contained acellular areas (eg, large areas of collagen, small folds, etc), or contained adnexal structures or large blood vessels, these areas were delineated with labelled annotations to be excluded from the automated digital analysis. Approximately 25 complete tissue spots were manually examined, and the tissue area of each tissue spot was calculated and averaged (approximately 320 000 µm^2^). The minimum tissue area was defined as the equivalent to approximately 30% of a complete tissue spot, or 100 000 µm^2^. The area of all tissue spots from a tumor sample were summed, and in order to be included in the analysis, the summed area was required to be equivalent to or greater than this minimum tissue area. The tissue cores were analyzed using the Tissue IA Optimiser program in SlidePath (Leica Microsystems) and the default DAB color definition. Algorithm settings were optimized for the Measured Stained Cells Algorithm (Suppl. Table S3); the algorithm quality control process is summarized in Supplemental Table S4, and representative image analyses are shown in Supplemental Figures S5 to S8. The algorithm selected for these analyses was designed to determine an H-score for each tumor. Although the H-score scoring system has limitations (eg, depending on the intensity and the percentage of positive cells, 2 tumors with different staining patterns may have identical H-scores), it is a commonly used scoring system in immunohistochemistry evaluation^9^ and was the scoring system available in the chosen software. An H-score cutoff of 80 was determined using X-tile plot software (version 3.6.1, Yale University School of Medicine, New Haven, CT) to stratify low- versus high-expressing beclin-1 tumors.

A Kruskal-Wallis test ANOVA with a Dunn post hoc test was used to compare beclin-1 H-scores. Kaplan-Meier functions and plots for DFI and ST were constructed with R statistical programming language (3.5.2) using the “survival” and “survminer” packages, and the logrank test was used to compare functions. Dogs lost to follow-up or those that died and whose cause of death was unrelated to the MCT were censored during statistical analysis. Cox proportional hazard ratios (HR) were calculated using the “coxphf” package (Cox regression with Firth’s penalized likelihood [version 1.13]), and the likelihood ratio was used to compare HRs. Differences were considered significant if *P* < .05.

## Results

### Grading and Mitotic Count

There were 37 cases with primary dermal MCTs that were not treated with adjunctive therapy and had outcome data, including 12 grade I tumors, 21 grade II tumors, and 4 grade III tumors based on Patnaik grading. The disease-specific median survival time (MST) for these cases was not reached for grade I tumors, was 1649 days for grade II tumors (95% confidence interval [CI] = 1530–NA), and was 119 days for grade III tumors (95% CI = 36–NA). There were statistically significant differences among Patnaik grades for both the MST (*P* < .0001) and disease-free intervals (*P* < .0001; Suppl. Figs. S9, S10).

There were 108 cases with primary dermal MCTs and primary subcutaneous MCTs that were not treated with adjunctive therapy and had outcome data, including 89 low-grade and 19 high-grade based on Kiupel grading. The MST was not reached in the low-grade tumors and was 211 days in the high-grade tumors (95% CI = 144–NA). The difference in survival between the 2 groups was significant (*P* < .0001; Suppl. Fig. S11), and dogs with a high-grade tumor were significantly more likely to die of MCT-related disease than dogs with a low-grade tumor (HR = 7.9, *P* < .0001). The difference in DFI between low- and high-grade tumors was also statistically significant (*P* < .0001; Suppl. Fig. S12), and dogs with a high-grade tumor were significantly more likely to have local recurrence or metastases than dogs with a low-grade tumor (HR = 6.0, *P* < .0001).

There were 71 primary subcutaneous tumors not treated with adjunctive therapy for which outcome data was known: 59 had a mitotic count of 4 or less in ten 400× fields (“low mitotic count”) and 12 had a mitotic count of 5 or greater in ten 400× fields (“high mitotic count”). The MST in this set of tumors was not reached in tumors with a low mitotic count and was 205 days in those with a high mitotic count (95% CI = 144–NA). The difference in survival between the 2 groups was significant (*P* < .0001; Suppl. Fig. S13), and dogs with a subcutaneous tumor having a high mitotic count were significantly more likely to die of MCT-related disease than those with a low mitotic count (HR = 8.2, *P* < .0001). The difference in DFI between low and high mitotic count was statistically significant (*P* < .0001; Suppl. Fig. S14), and dogs with a high mitotic count were significantly more likely to have local recurrence or metastases than dogs with a low mitotic count (HR = 6.2, *P* = .0001).

Comparing low mitotic count to high mitotic count dermal MCTs not treated with adjunctive therapy, the MST was not reached in tumors with a low mitotic count and was 119 days in those with a high mitotic count (95% CI = 36–NA). The difference in survival between the 2 groups was significant (*P* < .0001; Suppl. Fig. S15), and dogs with a high mitotic count were significantly more likely to die of MCT-related disease than dogs with a low mitotic count (HR = 76.8, *P* = .0002). The difference in DFI between low and high mitotic count was also statistically significant (*P* = .0002; Suppl. Fig. S16), and dogs having a tumor with a high mitotic count were significantly more likely to have local recurrence or metastases than dogs having a tumor with a low mitotic count (HR = 11.1, *P* = .004).

### KIT Immunolabeling Pattern

There were 90 cases of dermal MCTs and subcutaneous MCTs not treated with adjunctive therapy for which both outcome data was known, and for which the KIT immunolabeling pattern could be determined from the TMA. There were 38 pattern 1, 28 pattern 2, and 24 pattern 3 tumors. The disease-specific MST was not reached in patterns 1 and 2 and was 1710 days for pattern 3 tumors (95% CI = 673–NA). The overall difference in survival curves was statistically significant (*P* = .015; Suppl. Fig. S17). There was a significant difference between the hazard ratio of pattern 3 compared to 1 (HR = 3.0, *P* = .02) and 3 compared to 2 (HR = 3.6, *P* = .02), but not between patterns 1 and 2. The difference in DFI comparing the 3 patterns was also statistically significant (*P* = .01; Suppl. Fig. S18), and there was a significant difference between pattern 3 compared to 1 (HR = 2.9, *P* = .02) and pattern 3 compared to 2 (HR = 3.4, *P* = .01), but not between patterns 1 and 2.

### Beclin-1 Immunolabeling

**Figures 1–2.**
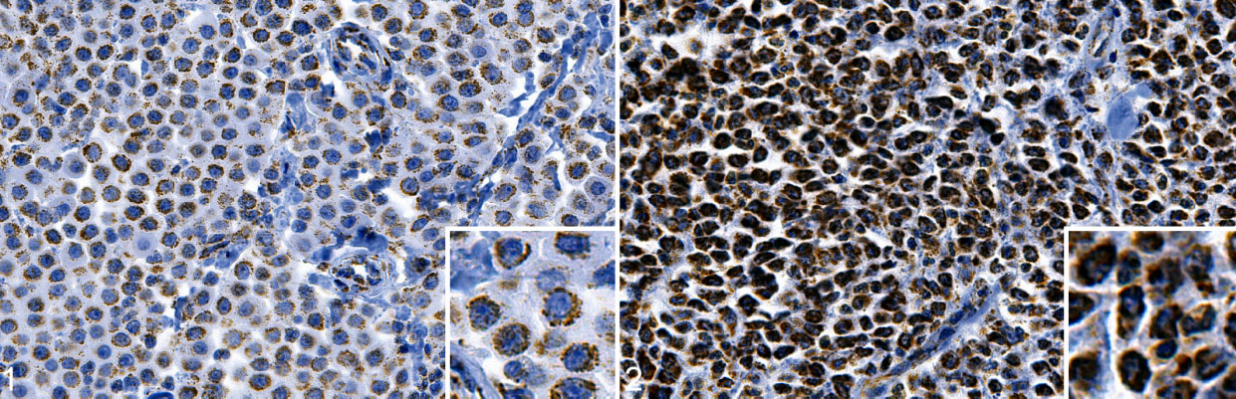
Mast cell tumor (MCT), skin, dog. Immunohistochemistry for beclin-1. **Figure 1.** An MCT with mostly weak to moderate granular cytoplasmic immunopositivity (low beclin-1 group). **Figure 2.** An MCT with mostly strong diffuse cytoplasmic immunopositivity (high beclin-1 group).

**Figure 3.**
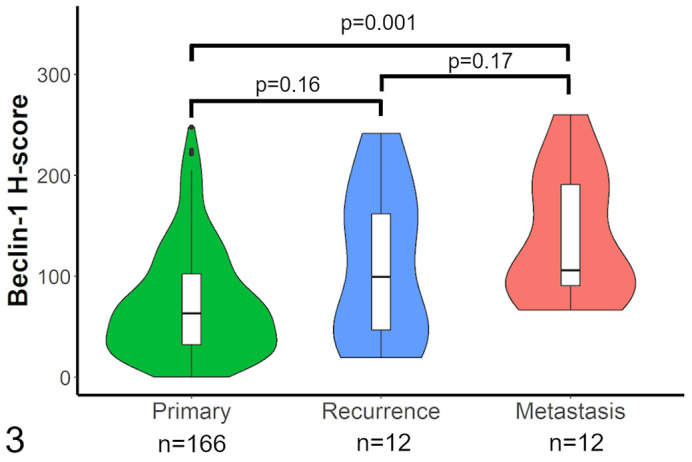
Violin plot of average beclin-1 H-scores in primary, recurrent, and metastatic canine dermal and subcutaneous mast cell tumors. The width of each plot shows the kernel probability density of the data (an approximation of the frequency of data points). The overlying box and whiskers plot displays the median, quartiles, and largest and smallest values that are at most 1.5 times the interquartile range. Outlier points are plotted individually as dots.

The immunolabeling of beclin-1 in MCTs was exclusively cytoplasmic, and varied from weak to strong in intensity, and from granular (usually perinuclear) to diffuse cytoplasmic in pattern (Figs. 1, 2 and Suppl. Figs. S19–S23). Beclin-1 immunolabeling was analyzed between primary, recurrent, and metastatic (lymph node only) canine dermal and subcutaneous MCTs. The beclin-1 immunolabeling was lowest in primary tumors (mean H-score = 73.5, *n* = 166), higher in recurrences (mean H-score = 110.8, *n* = 12), and highest in metastases (mean H-score = 138.5, *n* = 12; Fig. 3). There was a significant difference in means as calculated with a Kruskal-Wallis test (*P* = .0009), with a Dunn post hoc test showing a significant difference in means between primary and lymph node metastasis (*P* = .001), but not between primary and recurrence (*P* = .16) or recurrence and lymph node metastasis (*P* = .17).

### Beclin-1 as a Prognostic Biomarker in Untreated Tumors

To investigate the role of beclin-1 immunolabeling as a prognostic biomarker that informs on natural progression of MCT disease after surgical removal, beclin-1 levels in primary tumors not treated with adjunctive therapy were examined. Survival times for MCT-specific deaths and DFI for low versus high beclin-1 immunolabeling were examined in all primary dermal and subcutaneous MCTs not treated with adjunctive therapy. There was no significant difference in MCT-specific survival or DFI (Suppl. Figs. S24, S25). This result was the same when the MCTs were separated into dermal (Suppl. Figs. S26, S27) and subcutaneous (Suppl. Figs. S28, S29) MCTs.

To explore whether beclin-1 immunolabeling levels could be prognostic within certain subsets of MCTs that show different clinical behavior, outcome data were stratified by beclin-1 immunolabeling in different subsets: from dogs with low- and high-grade dermal and subcutaneous MCTs as defined by the Kiupel grading scheme; from dogs with KIT immunolabeling pattern I/II and pattern III dermal MCTs; from dogs with grade II and III dermal MCTs as defined by the Patnaik grading scheme (there were no events in those dogs with grade I MCTs); and from dogs with low and high mitotic count subcutaneous MCTs. No significant differences in the survival or disease-free curves were present (logrank *P* values are summarized in Suppl. Table S5).

### Beclin-1 as a Predictive Marker in Adjunctive Treated Tumors

**Figures 4–7.**
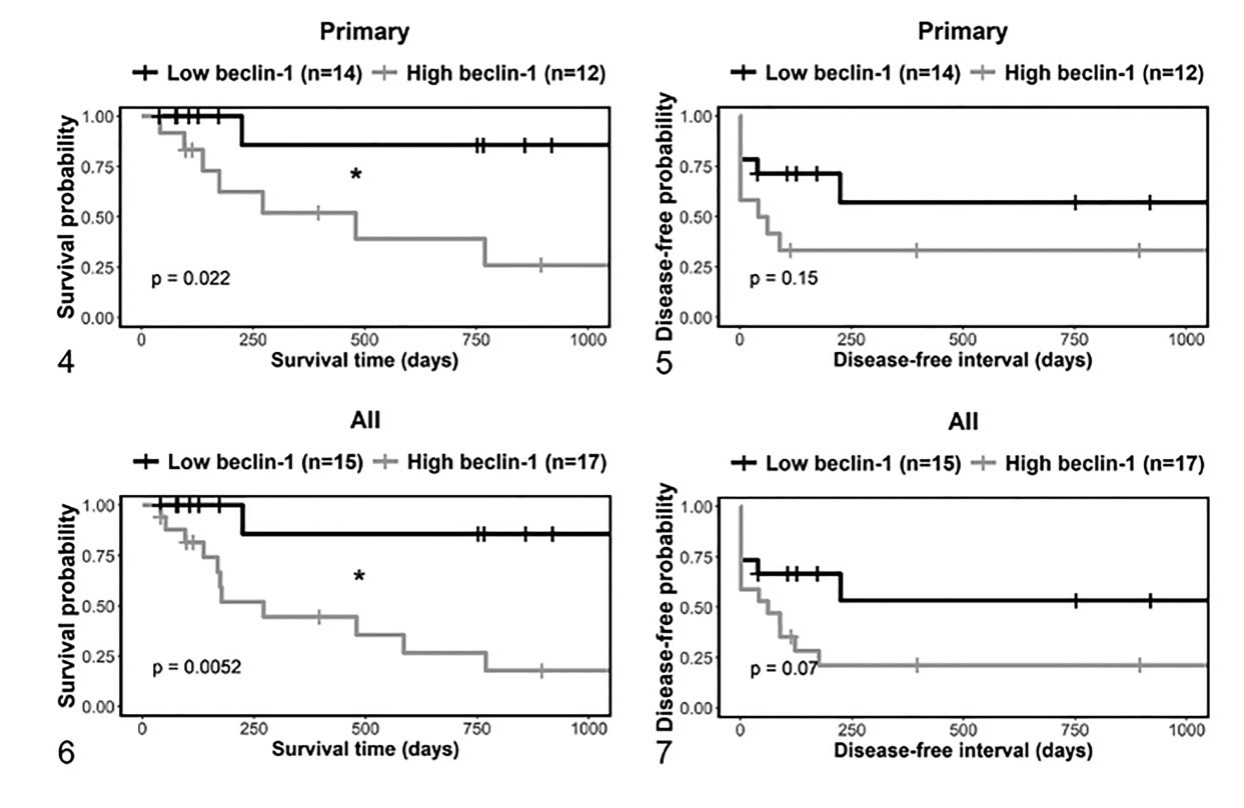
Kaplan-Meier survival curves for dermal and subcutaneous mast cell tumors that were treated with adjunctive therapy, stratified by beclin-1. Figures 4 and 6 show survival time, and Figures 5 and 7 show disease-free interval. Figures 4 and 5 include primary tumors only, and Figures 6 and 7 include primary and recurrent tumors. “All” refers to both primary tumors and recurrent tumors. The vertical tick-marks correspond to censored data. Survival functions were compared using the logrank test. *P < .05.

Some of the dogs received adjunctive treatment after surgical removal of the MCT (ie, treated with one or a combination of chemotherapy, radiation, or oral toceranib). The MCT-disease-related survival was significantly shorter for high compared to low beclin-1 immunolabeling, both in adjunctive therapy-treated dogs with primary MCTs (Figs. 4, 5) and for the combined data of dogs with primary or recurrent tumors (Figs. 6, 7). The disease-related survival was shorter for dogs with high compared to low beclin-1 expressing primary MCTs (HR = 5.7, *P* = .02, Cox proportional hazard analysis), and for the combined data of dogs with primary or recurrent tumors (HR = 7.4, *P* = .004). The difference in the DFI curves was not significant in either the primary MCTs (*P* = .15), or for the combined data of dogs with primary or recurrent tumors (*P* = .07). Next, the predictive value of beclin-1 immunolabeling for survival was analyzed separately in dogs with dermal and subcutaneous MCTs. The number of primary and recurrent dermal tumors that were treated with adjunctive therapy was relatively low, and there were no significant differences in survival between low and high beclin-1 immunolabeling groups (Suppl. Figs. S30, S31). However, all MCT-related deaths occurred in those dogs with high beclin-1 immunolabeling. Similarly, almost all MCT-related deaths occurred in those dogs with high beclin-1 immunolabeling subcutaneous MCTs (Suppl. Figs. S32, S33).

**Figures 8–11.**
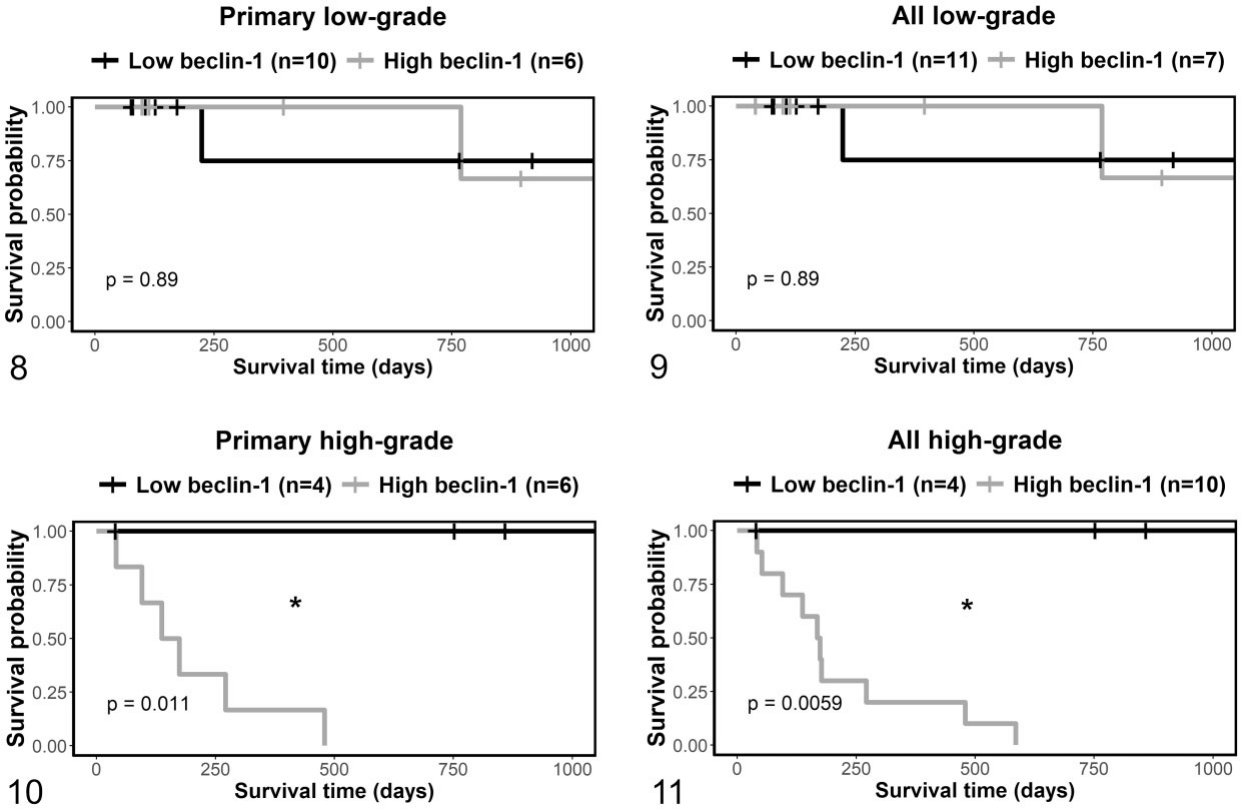
Kaplan-Meier survival curves for Kiupel low- and high-grade dermal and subcutaneous mast cell tumors that were treated with adjunctive therapy, stratified by beclin-1. Figures 8 and 10 show survival time, and Figures 9 and 11 show disease-free interval. Figures 8 and 9 include primary tumors only, and Figures 10 and 11 include primary and recurrent tumors. “All” refers to both primary tumors and recurrent tumors. The vertical tick-marks correspond to censored data. Survival functions were compared using the logrank test. *P < .05.

For dogs treated with adjunctive therapy for dermal or subcutaneous MCTs, those with Kiupel low-grade were then analyzed separately from those that were high-grade. There were no significant differences in survival in the low-grade subset of MCTs (Figs. 8, 9). In high-grade MCTs, there was a clear difference in survival outcome in dogs with primary MCTs (*P* = .011) and for the combined data of dogs with primary or recurrent tumors (*P* = .006; Figs. 10, 11). Within the high-grade MCTs, the survival was shorter for dogs with high compared to low beclin-1 expressing primary MCTs (HR = 16.3, *P* = .01, Cox proportional hazard analysis), and for the combined data of dogs with primary or recurrent tumors (HR = 18.9, *P* = .003).

**Figures 12–14.**
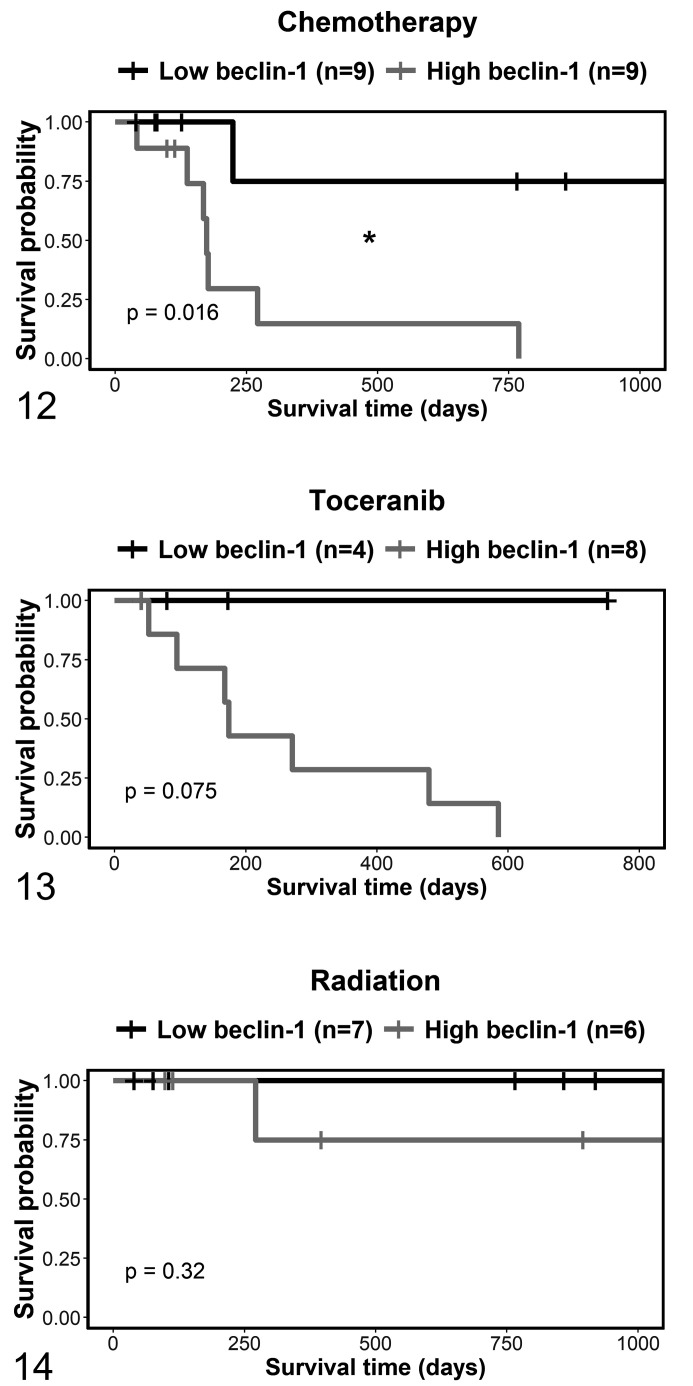
Kaplan-Meier survival curves for dermal and subcutaneous mast cell tumors treated with different adjunctive therapies, stratified by beclin-1. Primary and recurrent tumors are included. The vertical tick-marks correspond to censored data. Survival functions were compared using the logrank test. *P < .05.

There were 7 different possible combinations of adjunctive treatments, and the cases fell into 6 of these groups (Suppl. Table S6). Survival curves were analyzed from dogs with primary and recurrent skin MCTs that were treated with each of the 3 treatments, either including (Figs. 12–14) or excluding (Suppl. Figs. S34–S36) other treatment modalities. The disease-related survival was significantly shorter in dogs with high compared to low beclin-1 expression in chemotherapy-treated dogs that also had other treatment modalities (Fig. 12; *P* = .016), but not in dogs treated exclusively with chemotherapy (Suppl. Fig. S34; *P* = .15). Although not significantly different, the only MCT-related deaths occurred in the high beclin-1 immunolabeling group in the toceranib-treated dogs including other treatment modalities (Fig. 13; *P* = .075) and exclusively toceranib-treated dogs (Suppl. Fig. S35; *P* = .11). There were no significant differences in those dogs treated with radiation, with (Fig. 14) or without (Suppl. Fig. S36) other treatment modalities.

### Comparison Between Known Prognostic Markers and Beclin-1 Immunolabeling

To investigate the possible predictive value of known prognostic markers, survival functions in adjunctive-treated dermal and subcutaneous MCTs stratified by Kiupel grade, by mitotic count, and by KIT immunolabeling pattern were examined. There were significant differences in survival curves stratified by Kiupel grade (Suppl. Figs. S37, S38; primary, *P* = .005; primary and recurrent, *P* = .0006), mitotic count (Suppl. Fig. S39, S40; primary, *P* < .0001; primary and recurrent, *P* = .0001), and KIT pattern for primary and recurrent (Suppl. Fig. S42; *P* = .039), but not primary alone (Suppl. Fig. S41; *P* = .076). All the variables that showed apparent predictive ability in a univariable analysis were included in a multivariable analysis to determine which variable was most predictive of response to treatment (Table 1). In both primary-only adjunctive-treated dermal and subcutaneous MCTs (*P* = .041), and in primary combined with recurrent (*P* = .016), only beclin-1 immunolabeling levels had significant predictive value.

**Table 1.** Univariable and multivariable analyses of predictive factors for adjunctive treatment of combined primary and recurrent dermal and subcutaneous mast cell tumors^a^.

	Risk factor	Univariable			Multivariable		
		Hazard ratio	95% Confidence interval	*P* value	Hazard ratio	95% Confidence interval	*P* value
Primary AT- treated dermal and SC MCTs	High beclin-1 (vs low)	5.7	1.2–53.8	.024*	6.3	1.1–82.0	.041*
	MC >4 (vs MC ≤4)	7.2	1.9–27.2	.004*	8.1	0.1–191.5	.307
	High Kiupel grade (vs low)	4.8	1.5–19.3	.007*	1.6	0.1–251.3	.788
	KIT pattern III						
	(vs pattern I)	4.6	1.1–20.7	.036*	0.6	0.1–7.8	.623
	(vs pattern II)	2.6	0.6–15.1	.216	1.6	0.3–10.8	NA^b^
Primary and recurrent AT-treated dermal and SC MCTs	High beclin-1 (vs low)	7.4	1.7–68.0	.004*	6.8	1.4–70.9	.016*
	MC >4 (vs MC ≤4)	6.5	2.3–22.1	<.001*	10.1	0.1–211.3	.264
	High Kiupel grade (vs low)	5.9	2.0–22.7	<.001*	1.3	0.1–189.8	.855
	KIT pattern III						
	(vs pattern I)	3.9	1.2–13.8	.021*	0.4	0.1–3.1	.395
	(vs pattern II)	2.6	0.7–14.3	.158	1.2	0.3–6.4	.853

Abbreviations: AT, adjunctive therapy; MC, mitotic count; SC, subcutaneous.

^a^ Univariable analyses included all MCTs with datapoints that were available for each individual factor, whereas the multivariable analysis included only those MCTs with datapoints that were available for all factors.

^b^ NA denotes convergence in estimating profile likelihood *P* value could not be attained.

*P value < .05.

## Discussion

In this study, beclin-1, a protein involved in autophagy, was identified as a promising novel predictive biomarker in Kiupel high-grade tumors. Although mitotic count, KIT immunolabeling pattern, and Kiupel grade appeared to predict response to therapy in MCTs treated with adjunctive therapy, this apparent predictive ability was likely confounded by the fact that these are well-recognized prognostic factors, and MCT behavior can vary from benign to aggressive. In other words, some of the adjunctive-treated low-grade MCTs that appeared to have a good response to adjunctive treatment may in fact never have progressed regardless of whether treatment was given or not (hence falsely attributing predictive ability to the analyzed biomarker). In a multivariable analysis of these 4 factors, only beclin-1 immunolabeling maintained statistical significance. High beclin-1 versus low beclin-1 immunolabeling had a hazard ratio for MCT-specific deaths of 6.3 (*P* = .041) and 6.8 (*P* = .016), in primary-only adjunctive-treated dermal and subcutaneous tumors and in primary combined with recurrent, respectively. Therefore, beclin-1 immunolabeling level is the best independent predictive biomarker from this study, with high beclin-1 immunolabeling associated with higher MCT-specific deaths in dogs treated with adjunctive therapy.

This study relied on TMA technology to evaluate large numbers of MCTs in a high-throughput manner. There have been numerous validation studies to help address concerns about TMA technology. The most frequent concern about TMAs is whether a small tissue core accurately represents the entire tissue section.^14^ One validation study using human breast carcinoma found that two 0.6-mm cores were representative of tumor antigen expression in more than 95% of cases.^5^ In a study of Hodgkin lymphoma, two 0.6-mm cores had a concordance of 93.8% with whole-section analysis.^12^ Another study of human breast cancer found that a single 0.6-mm core was sufficient to identify statistically significant associations between receptor expression and clinical outcome.^45^ Another concern with immunohistochemistry studies in general is the effect of preanalytical variables, especially for retrospective studies. One example is the potential for loss of immunolabeling after prolonged storage of blocks, but one report found that many proteins showed successful immunoreactivity from samples stored for up to 60 years.^5^ Other examples of preanalytical variables include fixation delay, time in fixation, and conditions of histological processing. Future prospective studies are needed to confirm the results of this high-throughput, retrospective analysis.

To date, the only biomarkers examined as being potentially predictive for canine MCTs are ones that are known prognostic biomarkers for MCTs. One study examined c-*KIT* mutational status and its predictive effect in response to RTK inhibitor therapy.^24^ In this study, there was a response rate of 69% to toceranib in dogs with recurrent, Patnaik grade II or III MCTs with c-*KIT* mutations, whereas in those without mutations, there was a 37% response rate.^24^ Another study showed little difference in tumor response to masitinib between those MCTs with and without c-*KIT* mutations,^15^ while a recent study found similar results with toceranib treatment.^50^ It was hypothesized that these RTK inhibitors may not only be influencing unregulated KIT activity, but also activity of other RTKs such as VEGFR2 and/or PDFGRα/β in neoplastic and/or associated stromal cells that may be playing a role in tumor progression. As such, the mutational status of c-*KIT* has not been widely adopted as a clinically useful predictive marker, and if known, does not typically influence the decision to pursue RTK inhibitor therapy. Another recent study found that mitotic count, c-*KIT* mutation status, KIT localization, Patnaik histologic grade, and pKIT immunoreactivity were significantly associated with progression free interval in toceranib-treated dogs in a univariate analysis, while KIT localization and mitotic count retained significance in a multivariate analysis.^42^

Beclin-1 plays a key role in autophagy, a process that is increasingly recognized to be dysregulated in neoplasia. The role of autophagy in cancer is not straightforward, and is likely dependent on the microenvironment, stage of development, and neoplastic cell type. In some cases, beclin-1 appears to act as a tumor suppressor. For example, monoallelic deletion of the *BECN1* gene was found in several human cancers, and its loss is actually thought to contribute to tumorigenesis.^11,36,38^ In cell lines of gastrointestinal stromal tumors, a mutant KIT-driven cancer, knockdown of *BECN1* leads to accumulation of mutant KIT.^18^ In other cases, the upregulation of autophagy is thought to protect cancer cells and promote cancer growth in times of metabolic, hypoxic, and/or cytotoxic stress. Autophagy also plays a role in promoting cellular motility and invasiveness, which are necessary for tumor metastasis.^29^ The data of this study might suggest a protective role for beclin-1, perhaps allowing neoplastic cells to survive in the face of adjunctive therapy treatment.

Beclin-1 immunolabeling level was investigated as a prognostic and predictive marker in multiple human neoplasms,^8,17,23,33,53^ and its role as a prognostic marker appears to be dependent on the type of cancer. One meta-analysis found that a high beclin-1 immunolabeling level indicated a more favorable prognosis in gastric cancer and lymphoma, whereas it had no prognostic value in colorectal, breast, and lung cancers.^17^ Two studies investigating beclin-1 immunolabeling as a predictive marker found similar results to the current study. One found that high (vs low) beclin-1 immunolabeling in neoplastic rectal carcinoma cells was significantly associated with a reduced rate of tumor downstaging following neoadjunctive chemoradiation treatment.^53^ Another showed that patients with esophageal squamous cell cancer negative for immunolabeling of beclin-1 and microtubule-associated protein light chain 3 (LC3, another autophagy marker) had better overall survival. Furthermore, LC3 immunolabeling level was an independent predictive factor in patients receiving definitive chemoradiation.^6^

The role of autophagy in canine cancer is not well understood.^26^ In a panel of canine osteosarcoma cells, autophagy contributed to chemoresistance, and the autophagy inhibitor spautin-1, which enhances degradation of beclin-1, increased cell killing and decreased colony formation when combined with doxorubicin.^39^ In malignant canine mammary tumors, cytoplasmic beclin-1 immunolabeling was reported to be lower in neoplastic cells than in surrounding non-neoplastic mammary epithelial cells, and decreased cytoplasmic beclin-1 immunolabeling was significantly associated with poorer overall survival.^22^ Another study examining the protein P62/sequestosome-1 has suggested that autophagy may be important in MCT biology.^34^ The P62 protein is a “hub” protein that acts as an adaptor molecule to influence the uptake by autophagosomes of cargo targeted for autophagic degradation. Specifically, P62 nuclear and cytoplasmic immunoreactivity were associated with Kiupel low- and high-grade tumors, respectively.^34^ Although no outcome data were available for that study, these results raised the possibility that cytosolic P62 may be an indicator of increased autophagy in high-grade MCTs. In the present study, in contrast to the results in canine mammary carcinoma,^22^ beclin-1 immunolabeling level was a purely predictive factor and had no apparent prognostic value.

Although beclin-1 proved to be a significant predictive maker for response to adjunctive treatment in our dataset, there were limited numbers of treated dogs in each of the 7 different categories (each type of treatment alone, and in different combinations). This hindered our ability to identify exactly which treatment responses were being predicted. It is plausible that beclin-1 could be cytoprotective for all 3 modalities employed in the adjunctive treatment of canine MCTs. Conventional cytotoxic drugs are now well known to induce autophagy,^52^ but autophagy is also shown to help protect cancer cells against radiation therapy,^31^ as well as treatment with RTK inhibitors.^16^ Clinical trials are currently underway to investigate the effect of autophagy inhibitors, such as chloroquine and hydroxychloroquine, alone or in combination with chemotherapies in various human cancers.^30,52^ Phase I clinical trials for combined hydroxychloroquine and doxorubicin in dogs with lymphoma have also been completed.^2^ Thus, there is a potential that MCTs with high beclin-1 expression might yet respond to current therapies if delivered in combination with autophagy inhibitors.

The small number of adjunctive-treated dogs with tissue spots that met the inclusion criteria to derive an H-score for beclin-1 is a limitation in this study. Given that the results remained statistically significant in a multivariable analysis, and the results are reasonable in a biological sense, these data are promising. Another limitation of this study is the retrospective design, and inclusion of the MCTs in the TMA was based on tissue availability. A combination of non-adjunctive-treated and adjunctive-treated samples from primary and tertiary clinics resulted in lower overall numbers for each studied subgroup; yet, this combination allowed us to investigate the role of beclin-1 as both a predictive and prognostic marker. Although only skin MCTs removed with excisional biopsies were included in the analysis, incompletely excised MCTs were not excluded, as this would have eliminated the radiation-treated MCTs from our analysis. Additionally, many MCTs do not recur even when apparently incompletely excised, especially those MCTs that are expected to have better prognoses.^40,44,46^

Although many of the tumors included in the TMA were removed at a tertiary clinic, much of the follow-up data was obtained from the primary care veterinary clinics. A postmortem examination was not always performed, and the presence of local recurrence and metastasis was, in a small number of cases, based on physical rather than histological examination. Future investigation of the role of autophagy as a prognostic marker in canine MCTs should include a prospective analysis with higher numbers of adjunctive-treated dogs with long-term follow-up and postmortem examinations.

This study identified beclin-1 as a predictive marker that may prove useful in deciding whether to pursue adjunctive treatment after surgical removal of a skin MCT, especially for high-grade tumors. Predictive markers play a key role in personalized medicine, which is becoming increasingly important as the number of different treatment modalities grows. Clinicians and owners may have a relatively easy time deciding whether to pursue additional treatment for those MCTs with excellent prognosis (eg, 5-mm-diameter, excised with wide margins, grade I MCT) or very poor prognosis (eg, 6-cm-diameter, marginally excised, grade III MCT). However, the vast majority of dermal MCTs are grade II MCTs whose behavior is difficult to predict. In addition, there are many subcutaneous MCTs whose mitotic count is very close to the cutoff of 4, therefore complicating the decision on whether to pursue adjunctive therapy. Along with other factors that will always play a role in the decision, such as surgical margins, MCT location, owner finances, comorbidities, and anticipated side-effects, knowing the beclin-1 status to help determine whether the MCT has a good or poor chance of responding to treatment may help guide many clinicians and owners in making their choice. This study could prove useful in not only directing individualized treatment plans in dogs with MCTs but also in helping develop effective treatments for those dogs with nonresponsive tumors by combining current therapies with autophagy inhibitors.

## Supplemental Material

Supplemental Material, sj-pdf-1-vet-10.1177_03009858211042578 - Beclin-1 is a novel predictive biomarker for canine cutaneous and subcutaneous mast cell tumors Click here for additional data file. Supplemental Material, sj-pdf-1-vet-10.1177_03009858211042578 for Beclin-1 is a novel predictive biomarker for canine cutaneous and subcutaneous mast cell tumors by Britta J. Knight, Geoffrey A. Wood, Robert A. Foster and Brenda L. Coomber in Veterinary Pathology

Supplemental Material, sj-xlsx-1-vet-10.1177_03009858211042578 - Beclin-1 is a novel predictive biomarker for canine cutaneous and subcutaneous mast cell tumors Click here for additional data file. Supplemental Material, sj-xlsx-1-vet-10.1177_03009858211042578 for Beclin-1 is a novel predictive biomarker for canine cutaneous and subcutaneous mast cell tumors by Britta J. Knight, Geoffrey A. Wood, Robert A. Foster and Brenda L. Coomber in Veterinary Pathology
