# Supplementary material for: Beclin-1 is a novel predictive biomarker for canine cutaneous and subcutaneous mast cell tumors
Source: Vet Pathol. 2021 Sep 14;59(1):46–56. doi: 10.1177/03009858211042578 (PMC8679166; doi:10.1177/03009858211042578)
Supplement: Supplemental Material, sj-pdf-1-vet-10.1177_03009858211042578 - Beclin-1 is a novel predictive biomarker for canine cutaneous and subcutaneous mast cell tumors [file sj-pdf-1-vet-10.1177_03009858211042578.pdf]

Veterinary Pathology: Supplemental Materials.
Knight et al. Beclin-1 is a novel predictive biomarker
for canine cutaneous and subcutaneous mast cell tumors.

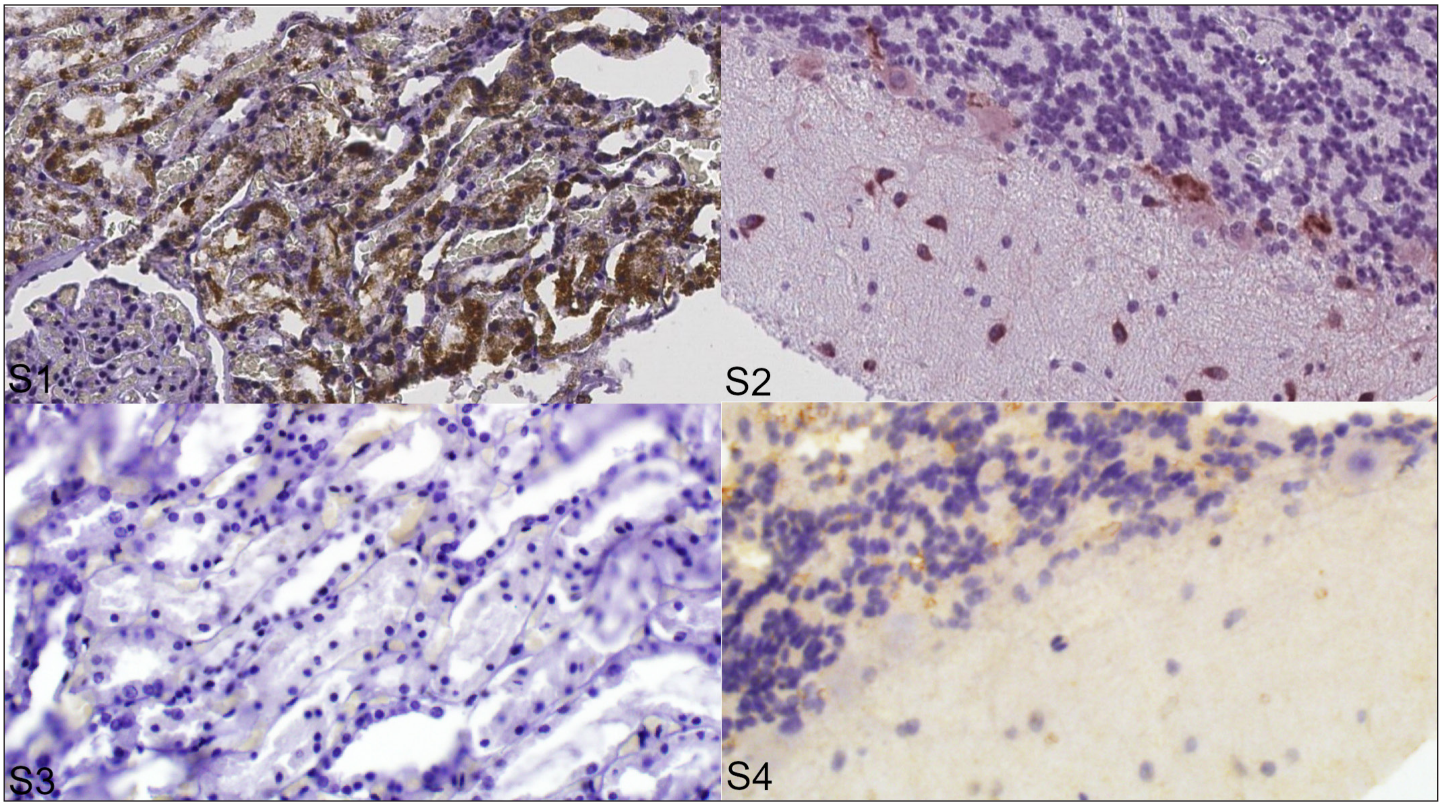

Supplemental Figures S1-S4. Positive and negative controls for immunohistochemistry. Figure S1. Renal tubular epithelial cells have weak to intense, granular to diffuse cytoplasmic positive immunoreactivity for beclin-1. Figure S2. Neurons in the cerebellum have moderate to intense, patchy to diffuse, cytoplasmic positive immunoreactivity for KIT. Figure S3. Renal tubular epithelial cells have no positive immunoreactivity using a mouse monoclonal IgG2a isotype control antibody. Figure S4. Neurons in the cerebellum have no positive immunoreactivity using a rabbit IgG, whole molecule control antibody.

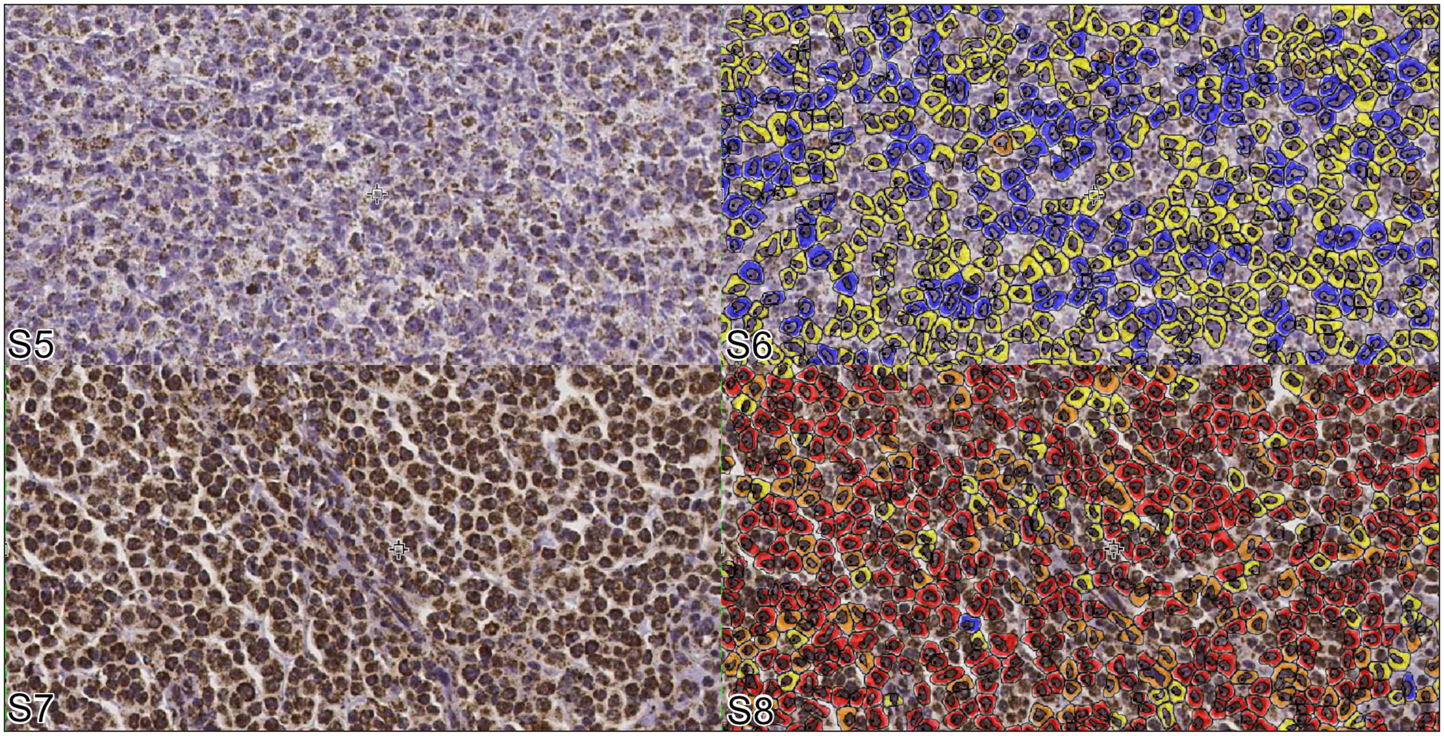

Supplemental Figure S5-S8. Immunohistochemistry staining patterns showing negative, low, medium, and strong cellular cytoplasmic immunopositivity of beclin-1. Two examples of MCTs, one with mostly negative/weak cytoplasmic immunopositivity (top), and one with mostly moderate/strong cytoplasmic immunopositivity for beclin-1 (bottom). The panels on the left (S5, S7) show the immunoreactivity for beclin-1, and the panels on the right (S6, S8) show the labeled cells after analysis with the automated image analysis software. For the labeled cells algorithm, the colors of the cells are as follows: Blue = negative; Yellow = weak; Orange = moderate; Red = strong.

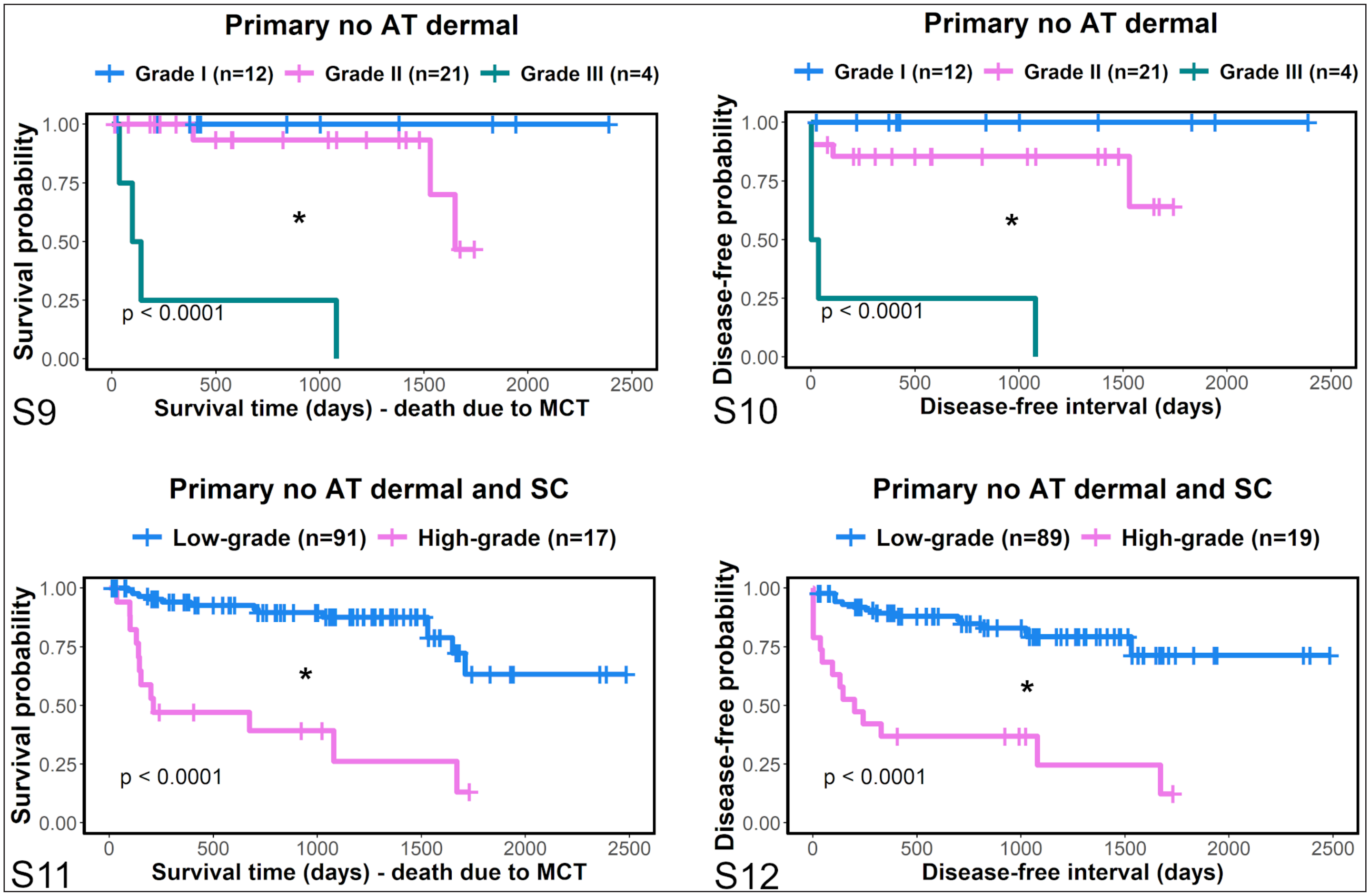

Supplemental Figures S9-S12. Kaplan-Meier survival curves for non-adjunctive therapy treated dermal mast cell tumors stratified by Patnaik and Kiupel grade. Figures S9,S11 show survival time and Figures S10,S12 show disease-free interval. The vertical tick-marks correspond to censored data. Survival functions were compared using the logrank test. AT, adjunctive therapy; MCT, mast cell tumour. *p<0.05

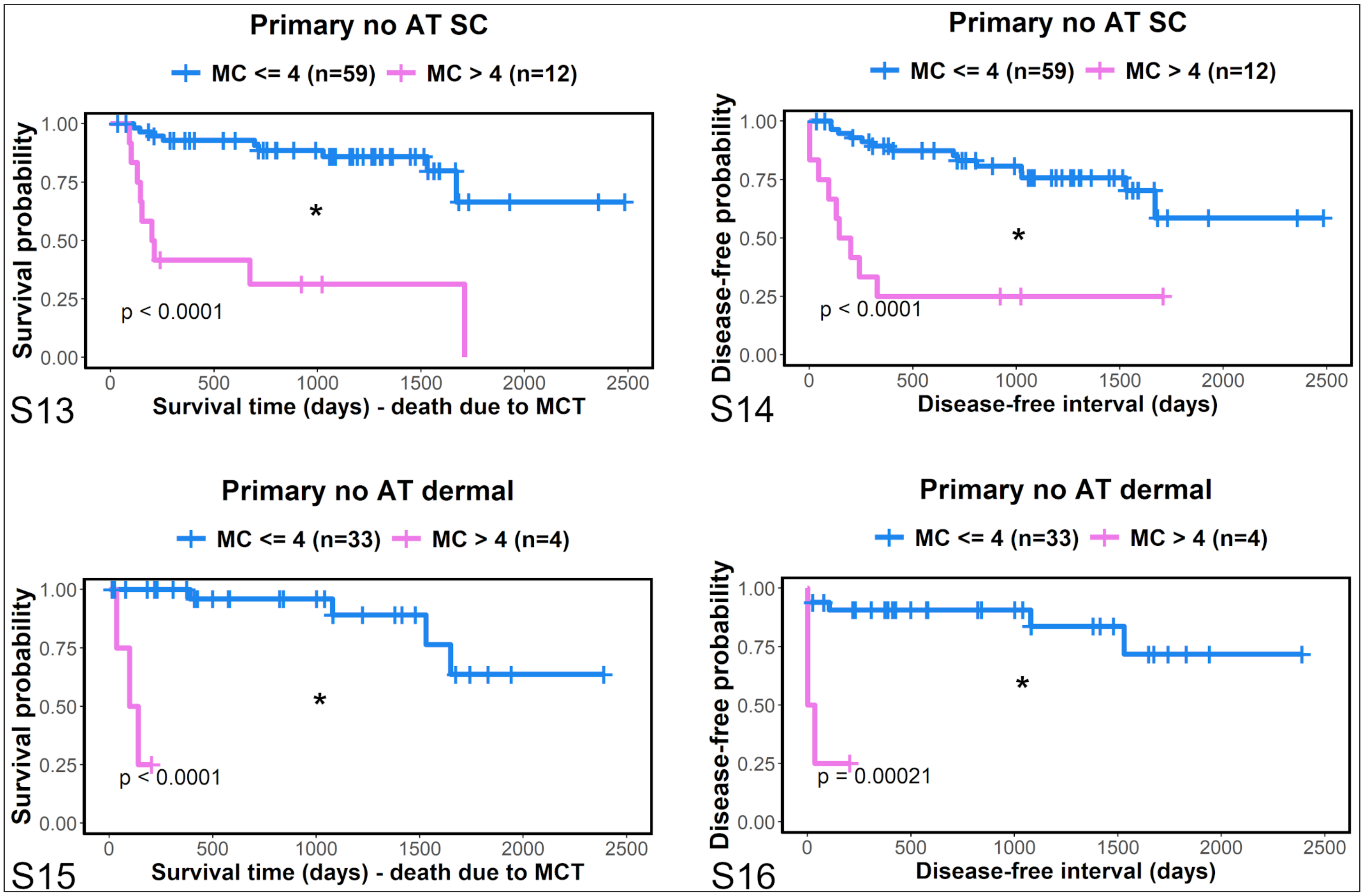

Supplemental Figures S13-S16. Kaplan-Meier survival curves for non-adjunctive therapy treated subcutaneous (S13,S14) and dermal (S15,S16) mast cell tumors stratified by mitotic count. Figures S13,S15 show survival time and Figures S14,S16 show disease-free interval. The vertical tick-marks correspond to censored data. Survival functions were compared using the logrank test. AT, adjunctive therapy; MC, mitotic count; SC, subcutaneous. *p<0.05

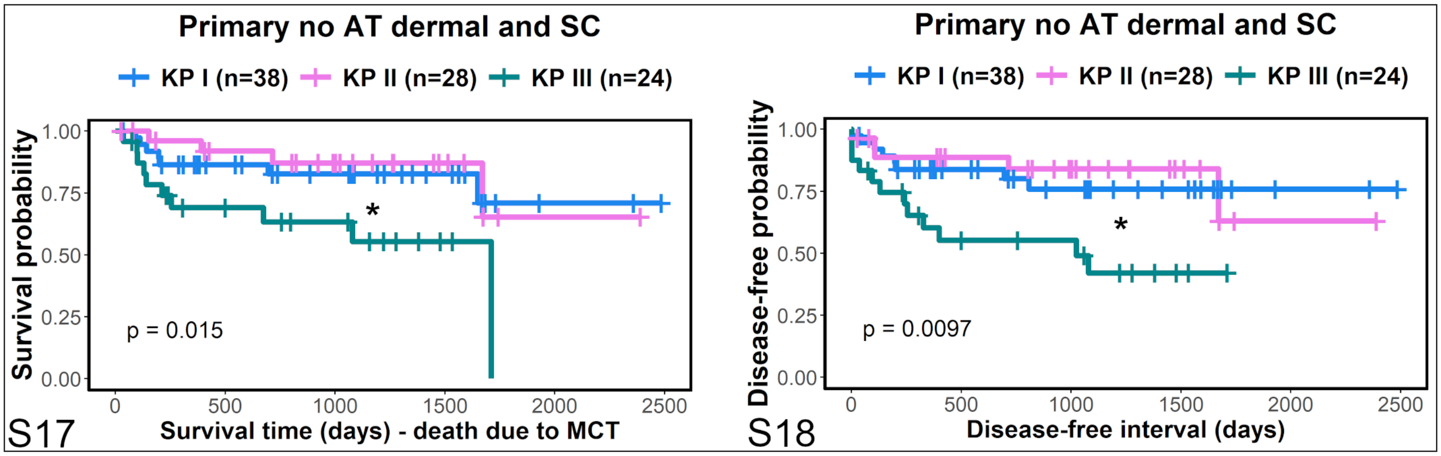

Supplemental Figures S17,S18. Kaplan-Meier survival curves for non-adjunctive therapy treated dermal and subcutaneous mast cell tumors stratified by KIT pattern. Figure S17 shows survival time and Figure S18 shows disease-free interval. The vertical tick-marks correspond to censored data. Survival functions were compared using the logrank test. AT, adjunctive therapy; SC, subcutaneous. *p<0.05

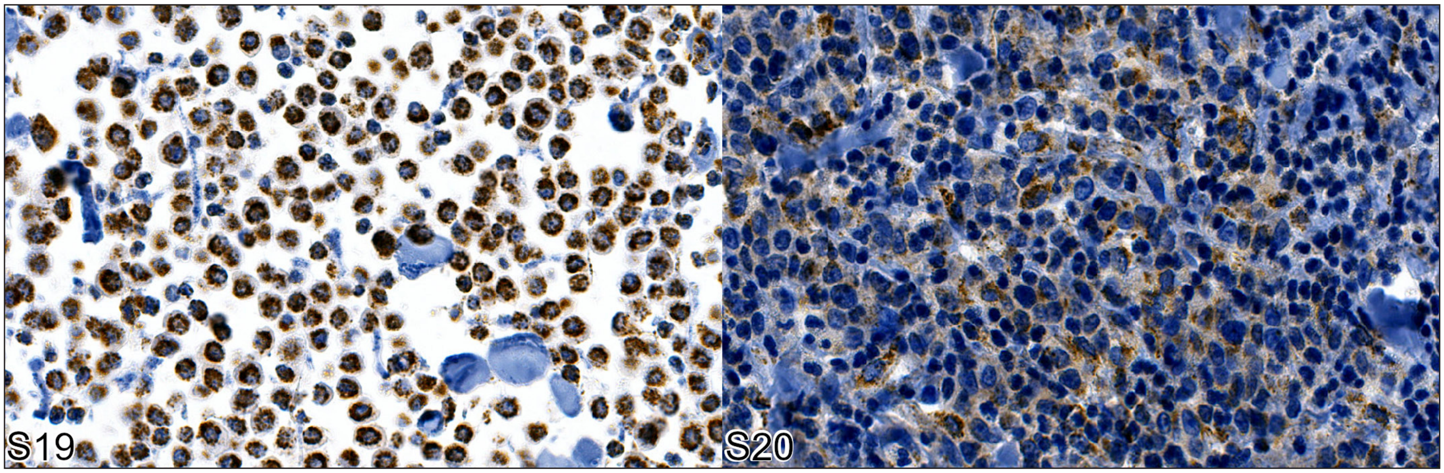

Supplemental Figures S19,S20. Examples of beclin-1 immunolabelling in different grades of tumors as defined by the Kiupel grading scheme. Figure S19. A low-grade tumor. Figure S20. A high-grade tumor.

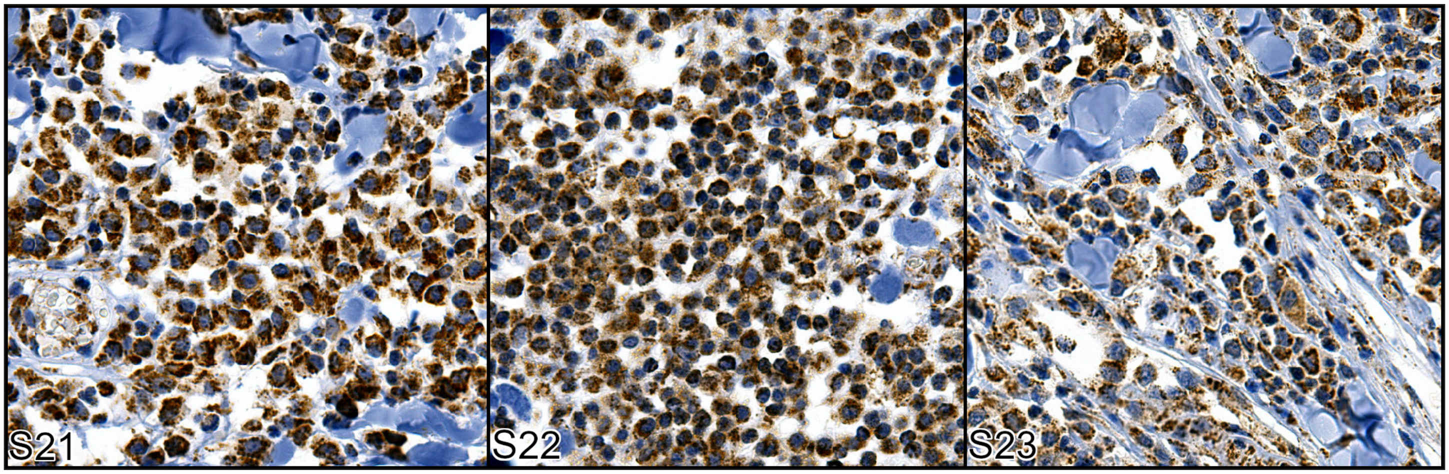

Supplemental Figures S21-S23. Examples of beclin-1 immunolabelling in different grades of tumors as defined by the Patnaik grading scheme. Figure S21. A grade I tumor. Figure S22. A grade II tumor. Figure S23. A grade III tumor.

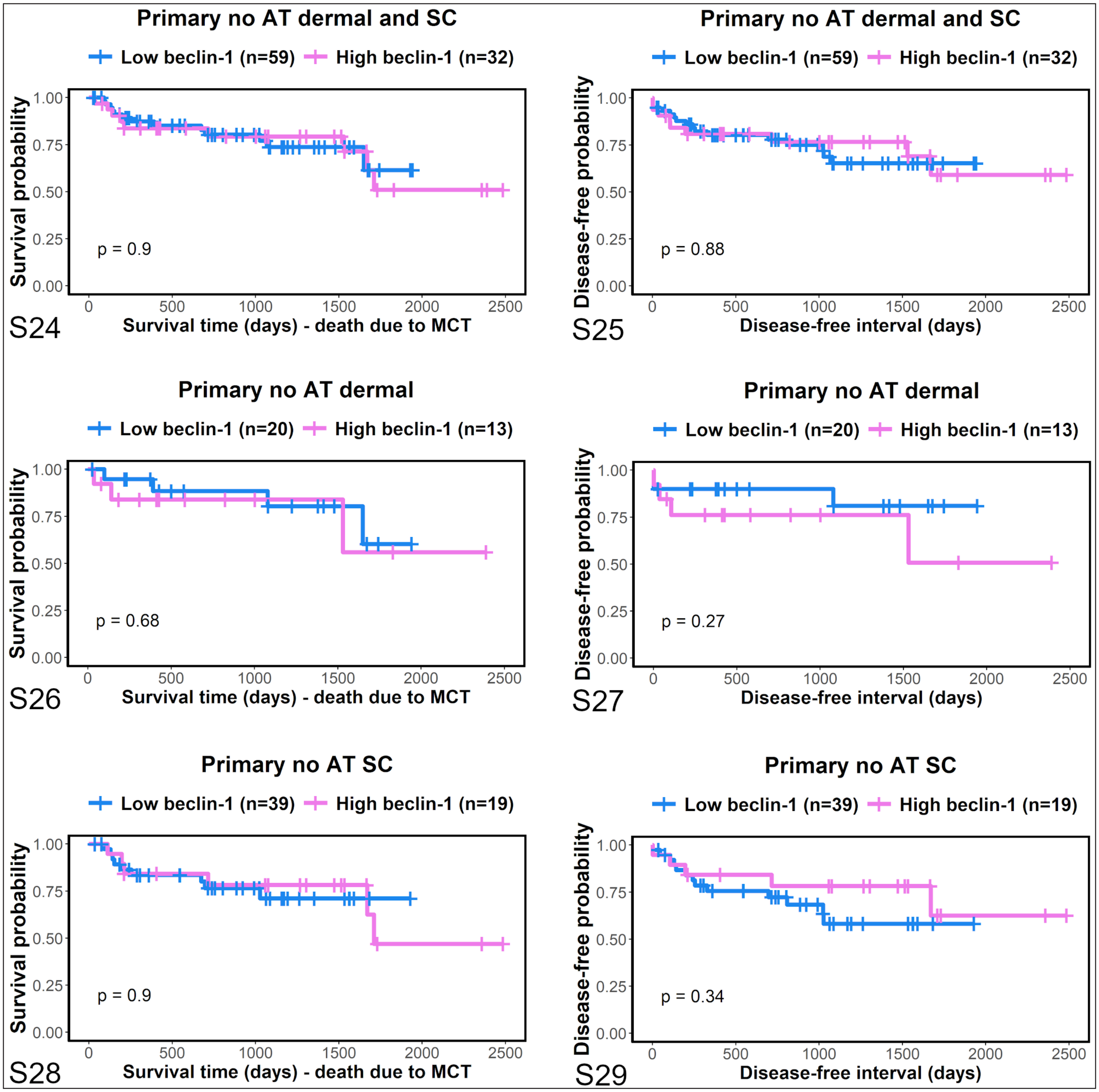

Supplemental Figures S24-S29. Kaplan-Meier survival curves for non-adjunctive therapy treated mast cell tumors stratified by beclin-1 staining. Figures S24,S26,S28 show survival time and Figures S25,S27,S29 show disease-free interval. The vertical tick-marks correspond to censored data. Survival functions were compared using the logrank test. AT, adjunctive therapy; SC, subcutaneous. $^*p<0.05$

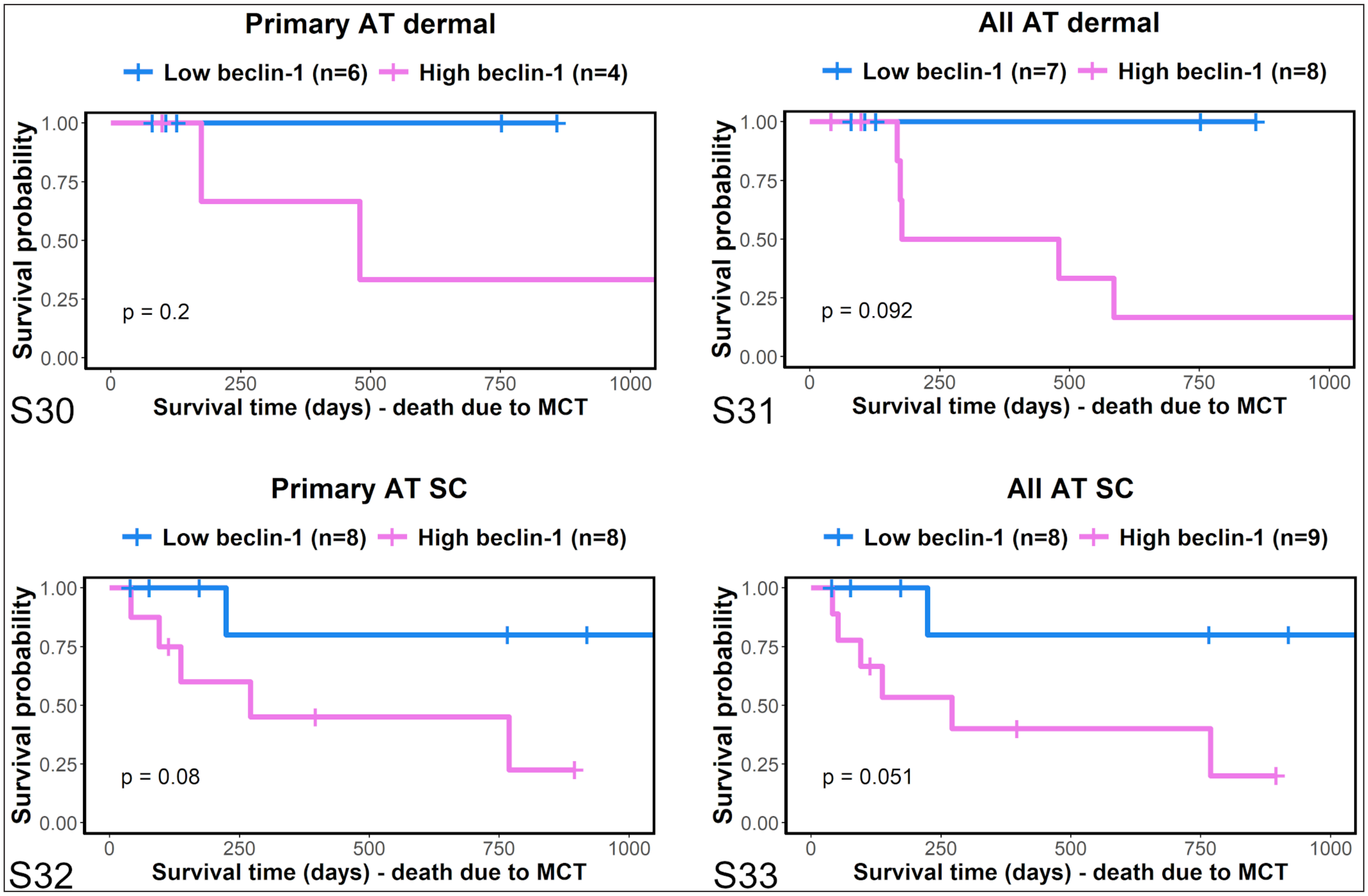

Supplemental Figures S30-S33. Kaplan-Meier survival curves for adjunctive therapy treated mast cell tumors stratified by beclin-1 staining. Figures S30,S32 include primary tumors only, and Figures S31,S33 include primary and recurrent tumors. The vertical tick-marks correspond to censored data. Survival functions were compared using the logrank test. AT, adjunctive therapy; SC, subcutaneous. *p<0.05

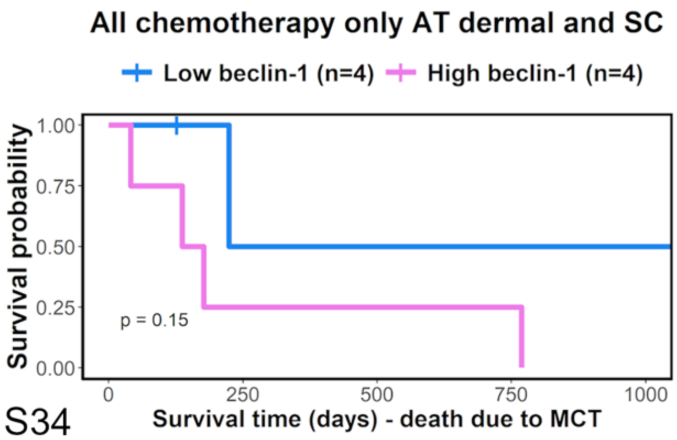

S34

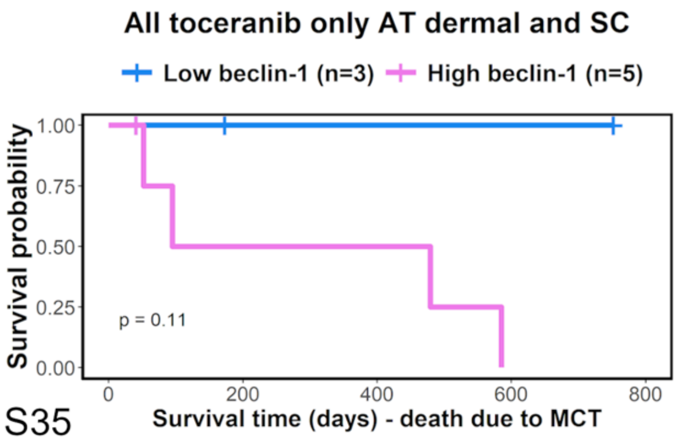

S35

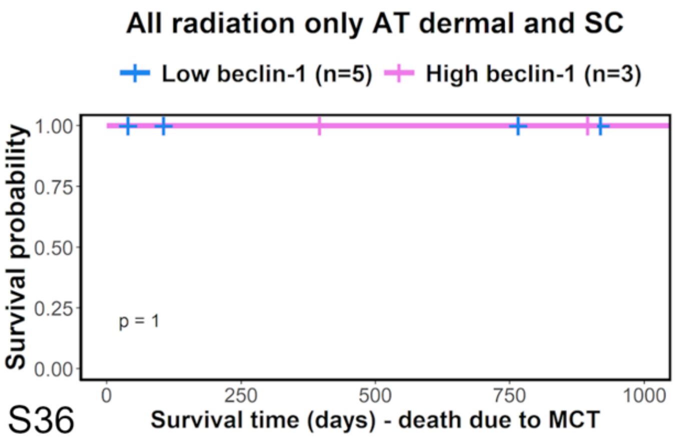

S36

Supplemental Figures S34-S36. Kaplan-Meier survival curves for single adjunctive therapy treated dermal and subcutaneous mast cell tumors stratified by beclin-1. Primary and recurrent tumors are included. The vertical tick-marks correspond to censored data. Survival functions were compared using the logrank test. AT, adjunctive therapy; SC, subcutaneous. *p<0.05

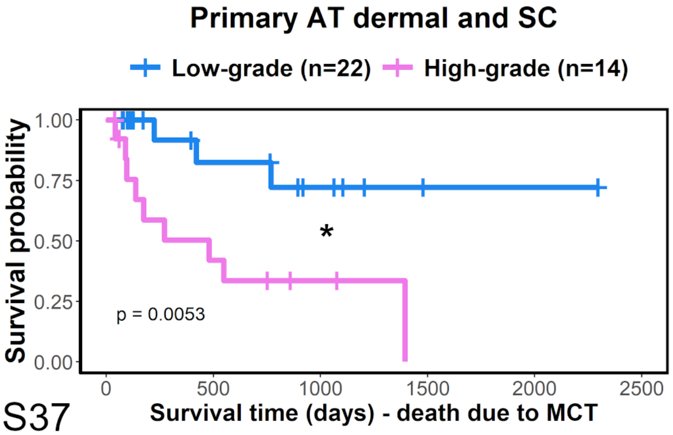

S37

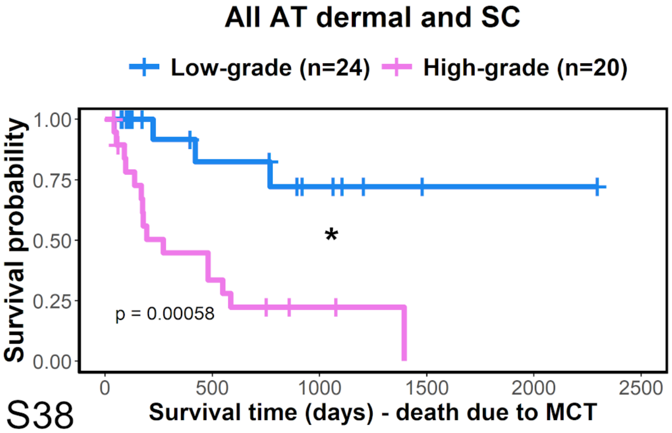

S38

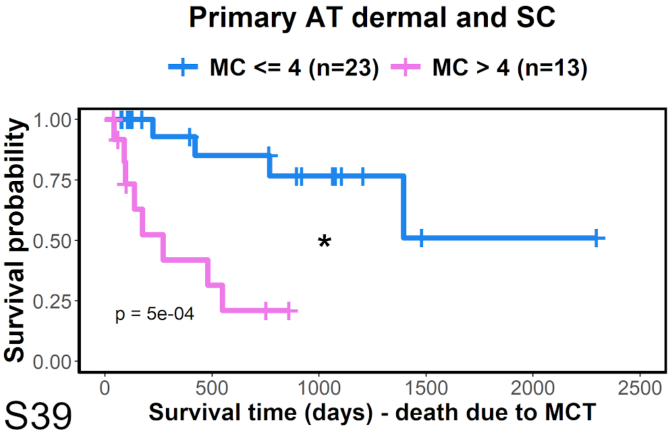

S39

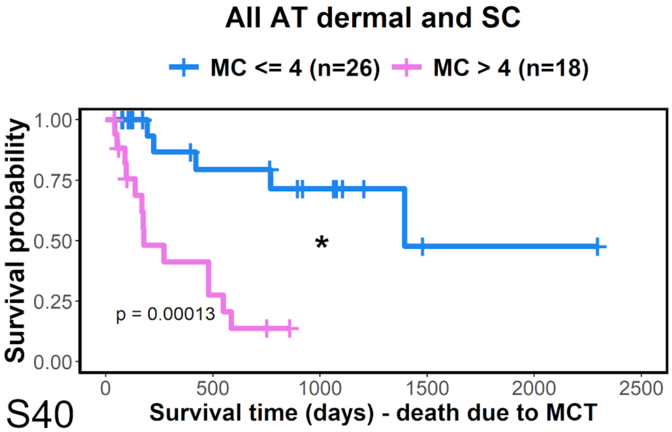

S40

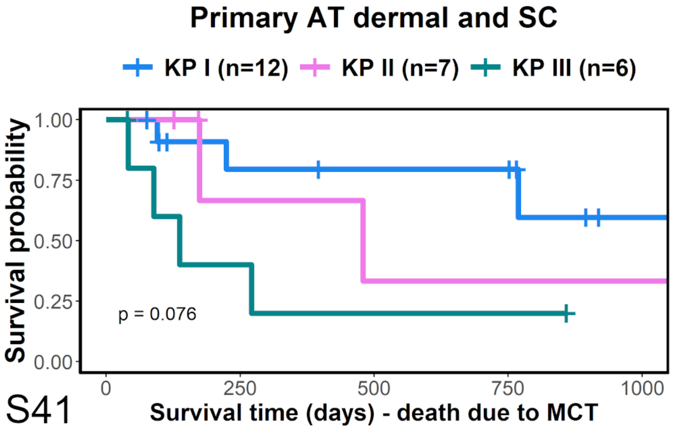

S41

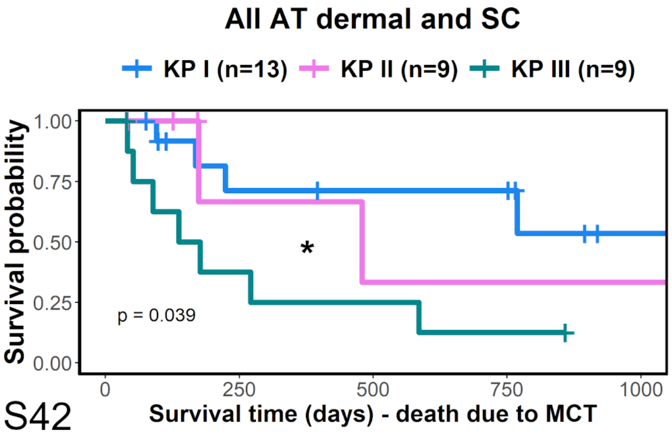

S42

Supplemental Figures S37-S42. Kaplan-Meier survival curves for adjunctive therapy treated dermal and subcutaneous mast cell tumors stratified by Kiupel grade (S37,S38), mitotic count (S39,S40), and KIT pattern (S41,S42). Figures S37,S39,S41 include primary tumors only, and Figures S38,S40,S42 include primary and recurrent tumors. The vertical tick-marks correspond to censored data. Survival functions were compared using the logrank test. AT, adjunctive therapy; SC, subcutaneous. *p<0.05

Supplemental Table S1. Summary of clinical data of dogs with mast cell tumors included in the tissue microarray. For dogs with multiple tumors, they were counted a single time per column in the ‘number of dogs’, ‘age’, ‘sex’, and ‘top breeds’ data.

	**Dermal**	**Subcutaneous**	**LN metastases**
Number of MCTs	110	106	13
Number of dogs			
With survival data	51	88	6
No survival data	31	15	5
Number of dogs (with survival data)			
Treated with AT	18	19	3
No AT treatment	33	69	3
Age (years)			
Mean (range)	7.5 (0.3 - 14.0)	7.9 (2.4 - 13.2)	9.5 (5.9 - 13.8)
Sex			
Male	5	4	1
Male neutered	24	40	4
Female	5	2	1
Female spayed	46	48	5
Unknown	2	4	0
Top breeds			
Labrador retriever	11	34	0
Mixed breed	15	20	1
Boxer	13	5	1
Golden Retriever	3	5	1
Type of tumor			
Primary	99	103	-
Recurrence	11	3	-
Metastasis	-	-	13
MCT source			
Biopsy	108	106	11
Post-mortem	2	0	2

AT, adjunctive therapy; LN, lymph node; MCT, mast cell tumor

Supplemental Table S2. Summary of outcome data of dogs with primary mast cell tumors included in the tissue microarray.

	**Dermal**	**Subcutaneous**
Number of primary MCTs with outcome	55	89
No MCT-related disease	35	57
Reported healthy at last follow-up	24	40
Death/euthanasia unrelated to MCT	11	17
MCT-related disease	20	32
Local recurrence	3 confirmed, 2 suspected	1 confirmed, 19 suspected
Metastasis	15	12
Lymph node metastasis	10 confirmed, 2 suspected	4 confirmed, 2 suspected
Disseminated metastasis	1 confirmed, 2 suspected	2 confirmed, 3 suspected
Unknown location of metastasis	-	1 confirmed
MCT-related death/euthanasia	13	24
Local recurrence	3 confirmed, 1 suspected	1 confirmed, 10 suspected
Metastasis	9	12
Lymph node metastasis	4 confirmed, 2 suspected	4 confirmed, 1 suspected
Disseminated metastasis	1 confirmed, 2 suspected	2 confirmed, 5 suspected
Local recurrence and disseminated metastasis	-	1 confirmed

MCT, mast cell tumor

Supplemental Table S3. Algorithm parameters for digital analysis of tissue microarray.

Input parameters	Settings	
Measurement units	0	
Tissue threshold	210	
Nuclei heterogeneity	2	
Strength of nuclear counterstaining	2	
Nuclear window radius size	37	
Nuclear area low/high threshold	25	150
Nuclei per window low/high threshold	10	500
% of nuclear area per window low/high threshold	10	100
Cell area low/high threshold	9	200
Maximum cell radius	80	
Cytoplasmic staining intensity cutoff	220	
% of cytoplasmic stained area in a cell cutoff	75	
Strong/moderate/weak cytoplasmic staining intensity cutoff	180	185

Supplemental Table S4. Algorithm quality control process.

<table>
<tr><th>Parameter</th><th>Quality control[1]</th></tr>
<tr><td colspan="2">Preanalytical variables</td></tr>
<tr><td>Tumor sampling for TMA</td><td>Every slide reviewed immediately prior to sampling of MCT block
Tumor area with highest cellular density was selected (potential for sampling bias)</td></tr>
<tr><td>IHC immunostaining</td><td>Both TMA blocks sectioned successively and both TMA slides immunostained in the same batch
Cores from control tissue blocks included in each of the TMA blocks
Immunostained slides reviewed for consistent immunostaining and counterstaining prior to whole slide image capture</td></tr>
<tr><td>TMA quality</td><td>Quality of unstained slides reviewed prior to selection for immunostaining
WSI scans reviewed for acellular areas (e.g. large areas of collagen, small folds, etc.), or non-MCT areas (e.g. adnexal structures, large blood vessels, etc.); these areas were delineated with labelled annotations to be excluded from the automated digital analysis
Poorly cellular areas (e.g. widely separated neoplastic mast cells) also excluded to ensure high cellular density of analyzed areas and excellent correlation between number of neoplastic mast cells and tissue area</td></tr>
<tr><td>WSI quality</td><td>All tissue spots reviewed for proper focus, consistent illumination and absence of artifacts (e.g. dust)
Any artifacts or poorly focused areas were delineated with labelled annotations to be excluded from the automated digital analysis</td></tr>
<tr><td colspan="2">Algorithm input parameters</td></tr>
<tr><td>Color definition</td><td>Default DAB color definition was selected to identify immunostaining positivity, and default haematoxylin color definition was selected to identify nuclear counterstain
Approximately 10% of tumor samples in each WSI reviewed by visual inspection to ensure adequate pixel readout</td></tr>
<tr><td>Neoplastic mast cell segmentation</td><td>Algorithm cellular/nuclear-related parameters were optimized to include the largest number of neoplastic mast cells (estimated >75% of mast cells were successfully segmented by visual inspection) while excluding the largest number of eosinophils, other cells, and non-cellular areas identified incorrectly as cells (estimated <2% of segmented cells were incorrectly segmented by visual inspection)
Approximately 50% of tumor sample tissue spots in each WSI reviewed by visual inspection during optimization of parameters</td></tr>
<tr><td>Classification of staining</td><td>Thresholds for staining intensity optimized to categorize the expression into four bins (negative, weak, moderate, strong) that correspond to the pathologist’s subjective classification of intensity (potential source of bias)
Approximately 50% of tumor sample tissue spots in each WSI reviewed by visual inspection during optimization of parameters</td></tr>
<tr><td colspan="2">Output review</td></tr>
<tr><td>Segmentation, binning, and H-score</td><td>All tissue spots reviewed by visual inspection for verification of segmentation (acceptance criteria [determined during optimization of segmentation] of approximately >75% of mast cells successfully segmented, approximately <2% of segmented cells incorrectly segmented), binning, and H-score
No tissue spots excluded at this stage</td></tr>
<tr><td>Tissue area</td><td>Minimum tissue area defined as approximately 30% of a complete tissue spot, or 100,000 μm^2
If the summed area of all tissue spots from a tumor sample was less than the minimum tissue area, the sample was excluded from the analysis</td></tr>
</table>

[1]All quality control steps conducted by a single pathologist (B.K.)

IHC: immunohistochemistry; MCT: mast cell tumor; TMA: tissue microarray; WSI: whole slide image

Supplemental Table S5. Summary of survival function p-values for mast cell tumors stratified by beclin-1 expression level. The *p*-values represent the logrank comparison between low and high beclin-1 expressing mast cell tumors for survival time and disease-free interval in different subsets of primary tumors not treated with adjunctive therapy.

	**Logrank test p-value**	
**Categories of primary MCTs not treated with AT**	**Survival**	**Disease-free interval**
Low-grade dermal and SC	0.72	0.93
High-grade dermal and SC	0.60	0.54
KIT I/II dermal and SC	0.82	1.00
KIT III dermal and SC	0.62	0.97
Grade II dermal	0.37	0.15
Grade III dermal	0.43	0.51
Low mitotic count SC	0.63	0.68
High mitotic count SC	0.61	0.78

AT, adjunctive therapy; MCT, mast cell tumor; SC, subcutaneous

Supplemental Table S6. Summary of number of dogs treated with different combinations of adjunctive therapies.

	**Primary**	**Recurrent**	**Totals**
**Chemo only**	7	1	**8**
**Rad only**	6	0	**6**
**Toc only**	4	4	**8**
**Chemo, rad only**	6	0	**6**
**Chemo, toc only**	2	1	**3**
**Rad, toc only**	0	0	**0**
**Chemo, rad, toc**	1	0	**1**
**Totals**	**26**	**6**	

Chemo, chemotherapy (vinblastine); Rad, radiation; Toc, toceranib

Supplemental Table S7 of individual-animal raw data is posted separately, as an Excel file.
